# Mitogenome of Endemic Species of Flying Squirrel, *Trogopterus xanthipes* (Rodentia, Mammalia) and Phylogeny of the Sciuridae

**DOI:** 10.3390/ani15101493

**Published:** 2025-05-21

**Authors:** Di Zhao, Zhongsong Wang, Wenyu Song, Wenge Dong

**Affiliations:** Yunnan Provincial Key Laboratory for Zoonosis Control and Prevention, Institute of Pathogens and Vectors, Dali University, Dali 671000, China; zhaodi5038@163.com (D.Z.); wangzs@sina.com (Z.W.)

**Keywords:** *Trogopterus xanthipes*, monotypic genus, Sciuridae, phylogeny, taxonomic status

## Abstract

*Trogopterus xanthipes* (Sciuridae, Rodentia) is a medium-sized flying squirrel species in the monotypic genus *Trogopterus*, and is endemic to China. It is distinguishable from other squirrels by the long black hairs on the inner and outer sides at the base of the ears and numerous ridges on the crowns of the upper and lower cheek teeth. Mitogenomes have been widely used in phylogenetic studies. We described *T. xanthipes* morphological features and successfully sequenced its mitogenome for the first time. The *T. xanthipes* mitogenome was conserved in number and order of genes. We analyzed codon usage patterns, evolutionary mutation rates, K2P distance, and genetic diversity of protein-coding genes. We reconstructed the phylogeny of Sciuridae (94 species and 21 genera in 4 subfamilies). All phylogenetic trees shared the same topologies and consistently supported the monophyly of Sciuridae, and the supported subfamilies relationship as follows: ((Xerinae + Callosciurinae) + Sciurinae) + Ratufinae. The relationship within the Sciurinae clade was ((*Glaucomys* + *Hylopetes*) + ((*Trogopterus*+*Pteromys*) + *Petaurista*) + *Sciurus)*. The relationship within the Callosciurinae clade was *Exilisciurus* + ((*Tamiops* + *Dremomys*) + ((*Lariscus*+*Sundasciurus*) + *Callosciurus*)). The relationship within the Xerinae clade was *Sciurotamias* + (*Tamias* + (*Callospermophilus* + (*Marmota* + (*Spermophilus* + (*Urocitellus* + (*Ictidomys* + *Cynomys*)))))). The phylogenetic position among different subfamilies of Sciuridae was consistently recovered with high support across different datasets (PCGRNA and PCG12RNA) and supported the monophyletic lineage of each genus of Sciuridae. *Trogopterus xanthipes* was sister species to *Pteromys volans.* Species within the genus formed different minor clades, suggesting relatively high interspecific divergences. The tribe Pteromyini was sister taxon of the tribe Sciurini, which was not supported by the traditional division of Sciuridae into subfamilies Pteromyinae and Sciurinae. Hence, our data supported a division of the Sciuridae into five subfamilies.

## Simple Summary

*Trogopterus xanthipes* is a near-threatened squirrel-like small rodent in the monotypic genus *Trogopterus* endemic to China. Mitogenomes have been widely used in phylogenetic studies. We described *T. xanthipes* morphological features, successfully sequenced and analyzed its mitogenome for the first time. We reconstructed the phylogeny of Sciuridae (94 species and 21 genera in 4 subfamilies). All phylogenetic trees shared the same topologies and consistently supported the monophyly of Sciuridae across different datasets (PCGRNA and PCG12RNA). Each genus of Sciuridae was monophyletic lineage. *Trogopterus xanthipes* was sister species to *Pteromys volans.* Species within the genus formed different minor clades, suggesting relatively high interspecific divergences. The tribe Pteromyini was sister taxon of the tribe Sciurini, which did not support the traditional division of Sciuridae into subfamilies Pteromyinae and Sciurinae. The presented mitogenomic phylogeny supports the division of Sciuridae into five subfamilies.

## 1. Introduction

*Trogopterus xanthipes* (complex-toothed flying squirrel) (Milne-Edwards, 1867) is a medium-sized species of the monotypic genus *Trogopterus* endemic to China [1,2,3]. It is listed as a near-threatened (NT) species in the IUCN Red List [1]. It is distinguishable from other squirrels by tufts of long black hair on the inner and outer sides of base of the ears and numerous ridges on the crowns of the upper and lower cheek teeth. Extensive modification and loss of suitable habitat for *T. xanthipes* due to logging, hunting, medical research, or pet trade, has led to the decline in the number of *T. xanthipes*, and the current population trend is unknown.

Sciuridae is increasingly viewed as a model clade for evolutionary and genetic studies because of their broad distribution, large variety of species (a total of 292 species and 60 genera in 5 subfamilies), small body size, short generation lengths, diversity of life-history traits, behavioral variation, ease of genetic sampling, and the precarious conservation status of many species. *Trogopterus xanthipes* belongs to the Pteromyini of Sciurinae, Sciuridae. The taxonomic status of the tribe Pteromyini has been controversial, as opinions have varied about whether it represents a distinct family or perhaps Pteromyini should be a subfamily. Pocock (1923) [4] divided squirrels and flying squirrels into two separate families (Sciuridae and Pteromyidae) based on penile features. Bruijn and Unay (1989) [5] separated squirrels and flying squirrels into two families based on tooth structure of the fossils of squirrels and flying squirrels from the Oligocene. Ellerman (1940) [6] proposed that squirrels and flying squirrels were divided into the *Pteromys* Group and the *Sciurus* Group based on the absence of glide membrane from forefoots to hindfoots. Elerman and Morrison-Scott (1951) [7] also classified the *Pteromys* with glide membrane into the Pteromyini. Hoffmann et al. (1993), Huang Wenji et al. (1995) and Wang Yingxiang (2003) all showed that squirrels and flying squirrels should be separated into two subfamilies (Sciurinae and Petauristinae) based on morphology [8,9,10]. With the development of molecular biology, more and more researchers have begun to integrate traditional morphology and phylogenetics, and applied them to taxonomy, population genetics, evolution, and phylogeny of the Sciuridae. The most representative research result was that Mercer and Roth (2003) [11] and Steppan et al. (2004) [12] classified Sciuridae into five subfamilies (Sciurillinae, Ratufinae, Callosciurinae, Sciurinae, and Xerinae), and the tribe Sciurini and Ptermoyini are grouped under the Sciurinae based on fossil and DNA data. In *Mammal Species of the World*, 3rd edition (2005), *A Guide to the Mammals of China* (2009), and *Taxonomy and Distribution of Mammals in China* (2022) also divided Sciuridae into five subfamilies [3,13,14], and the tribes Sciurini and Ptermoyini were grouped under the Sciurinae. Herron MD et al. (2004) [15] reconstructed phylogenetic relationships of the Sciuridae based on *CYTB* sequences of 114 species from 21 sciurid genera and found the phylogeny of squirrels to be in substantial conflict with the current taxonomy. Morphological and molecular data have greatly contributed to advances in the phylogeny and evolutionary dynamics of the Sciuridae. Although the Sciuridae has been well-supported by both molecular and morphological data, the relationship among subfamilies and interspecific divergences in major clades are not fully resolved. In this study, we used complete mitogenome sequences of scuirid taxa to further clarify the evolutionary relationships within the Sciuridae.

The mitogenome has often been used in systematics and phylogenetic studies across various taxonomic levels of different animals due to its nature of maternal inheritance, lack of recombination, simple structure, rapid evolutionary rate, and rich genetic information [16]. The highly conserved gene order of mitogenomes provides clear homology over hundreds of millions of years, while rapid mutation rates generate strong phylogenetic signals over short time frames, and yet, sites under strong purifying selection have very low substitution rates. Together, these features provide a uniquely wide domain of utility, making mitochondrial sequences attractive as phylogenetic markers—from populations through to phyla [17]. The selective sweeps and paucity of recombination that enhance the potential of mitogenomes as speciation indicators also render them susceptible to introgression in association with fitness differences across populations. Thus, high throughput sequencing of multiple loci diminishes the value of shallower mtDNA coalescence, by offering a far more extensive examination of species histories in the future. For the past decade, numerous studies have especially focused on sequencing and using complete mitogenomes rather than a single gene or partial sequences of mitochondrial DNA in exploring phylogenetic relationships in mammals [18,19,20,21,22]. When compared with short or highly conserved mitochondrial sequences at the locus level, complete mitogenomes are more useful and informative [23,24,25]. Mitogenomes provided molecular datasets that were sampled broadly enough to address such deep-level relationships between placental mammal orders. They were enthusiastically embraced and provided strong support for many now accepted relationships among placental mammals [26,27]. In recent years, mitogenomes have been widely used in evolutionary studies of sciurids. Boukhdoud et al. (2021) [28] reconstructed the phylogeny of Sciuridae based on the mitogenomes of 37 species of three subfamilies. Ibis et al. (2025) [29] presented evolutionary and taxonomic implications within the suborder Sciuromorpha based on complete mitochondrial genomes and *CYTB* genes, and provided new insights into the evolutionary relationships of mitochondrial lineages within the families Gliridae and Sciuridae. Mitogenomic phylogenetic analysis helps to increase our understanding of how species evolved. To address phylogenetic relationships and evolution of Sciuridae, we sequenced the *T. xanthipes* mitogenome and reconstructed the phylogeny of Sciuridae by combining the mitogenome sequences of 93 species from 20 genera and 4 subfamilies from the GenBank database. This study, the largest mitochondrial phylogenetic analysis of Sciuridae to date, highlights not only the usefulness of mitogenomic data for Sciuridae phylogeny, but also for characterizing trait diversification in Sciuridae evolution.

## 2. Materials and Methods

### 2.1. Specimen Collection and Identification

*Trogopterus xanthipes* was collected at elevations of 2500 m in Weixi Lisu Autonomous County, Yunnan (27.3256° N, 99.0416° E). Body mass (g), head–body length (mm), tail length (mm), hindfoot length (mm), and ear length (mm) were measured in the field. Specimen identification was mainly based on morphology, including body size, pelage color, cranium, and mandible. Species name and identification were referenced with *Taxonomy and Distribution of Mammals in China* [14] and *Handbook of the Mammals of the World* [1]. Individuals with intact cranium and good external morphology were selected as voucher specimens. We collected one male specimen and used it as a voucher specimen. An appropriate amount of muscle, liver, and spleen tissues were clipped and placed in Eppendorf tubes with 95% ethanol (containing a label with a unique reference code) and stored in the ultra-low temperature refrigerator at −80 °C until further processing. The specimens were stored in the Institute of Pathogens and Vectors, Dali University (Dali, China), the voucher number E07009. The Local Ethical Committee of Laboratory Animal Experimentation at Dali University approved all experiments involving *Trogopterus xanthipes*.

### 2.2. DNA Extraction, Mitogenome Sequencing and Analysis

The muscle tissue of one male *T. xanthipes* was removed from Eppendorf tube containing 95% ethanol and thoroughly shredded with sterile surgical scissors. Genomic DNA of *T. xanthipes* was extracted using a DNeasy Tissue kit (QIAGEN, Redwood City, CA, USA) following the manufacturer’s protocol. DNA was shredded into random fragments of 300–500 bp using a Covaris M220 Focused Acoustic Shear (Covaris, Inc., Woburn, MA, USA.). After measuring the purity and integrity of the amplified DNA, a DNA library was constructed. Sequencing was performed using the Illumina Novoseq 6000 platform (Winnerbio, Shanghai, China). At the same time, the *CYTB* barcode was sequenced for verification. Raw read quality was assessed with fastq 0.20.1. Raw data were filtered to obtain clean data (the GC content was 45.38%, Q20 value was 99.43%, and the Q30 value was 98.15%). MitoZ 2.3 [30] was used to assemble the clean data. BWA v0.7.17 [31] and Samtools v0.1.20 [32] were used to assess the confidence of the assembly data (sequencing depth ≥ 100×). Geneious Prime11.1.5 [33] was used to verify the accuracy of the assembly results. Contigs were de novo assembled from Illumina sequence reads using Geneious based on *COX1* and *rrnS* relatively conserved sequences. The assembly parameters were minimum overlap 150 bp and minimum overlap identity 99~100%. Protein-coding genes and rRNA genes were identified by BLAST 2.16 [34] and MITOS2 Web Server (accessed on 5 January 2025) [35], tRNA genes were identified by tRNAscan-SE 2.0 [36], and ARWEN 1.2 [37], and then manually checked and corrected. The annotated mitogenome sequence of *T. xanthipes* has been deposited in GenBank (accession number: PQ280108). Nucleotide composition and codon preference were analyzed using cowdonW. Skew was calculated as AT-skew = (A − T)/(A + T) and GC-skew = (G − C)/(G + C). DnaSP v6 [38] was used to analyze the evolution rate of 13 protein-coding genes (Nonsynonymous substitution rate (Ka), synonymous substitution rate (Ks)), and nucleotide diversity (Pi). MEGA 11 [39] was used to calculate Kimura 2-parameter (K2P) genetic distances of 13 protein-coding genes. Parity rule 2 (PR2), ENC-Plot (Effective Number of Codon, Nc), Neutrality curve and relative synonymous codon usage (RSCU) were analyzed using RStudio v4.3.1. The mitogenome circular map was drawn with Proksee [40].

### 2.3. Phylogenetic Analysis

Phylogenetic trees of 94 species representing 21 genera of 4 subfamilies in Sciuridae were constructed. *Glis glis* (Linnaeus, 1766), *Castor fiber* (Linnaeus, 1758), and *Ochotona curzoniae* (Hodgson, 1857) were used as outgroups (Species information: Table S1). Two concatenated alignments were used in subsequent phylogenetic analyses: (i) PCGRNA matrix, which contains all of the three codon positions of 13 protein-coding genes and the 2 rRNA genes and (ii) PCG12RNA matrix, which contains only the first and the second codon positions of 13 protein-coding genes and the 2 rRNA genes. All matrices were analyzed using maximum likelihood (ML) and Bayesian inference (BI) in the PhyloSuite v1.2.3 [41,42]. Multi-sequence alignment was performed using MAFFT v7.313 [43] and optimized with MACSE v2.06 [44]. Large gaps and ambiguous sites in protein-coding genes and rRNA sequences were modified using Gblocks 0.19b [45] and trimAI v1.4.rev15 [46]. The best-fit nucleotide substitution model for the tree was determined using ModelFinder v2.2.0 [47]. IQ-TREE v2.2.2.7 [48] was used to construct the ML tree and the number of bootstrap repetitions was set to 1000. The best-fit model was determined by ModelFinder and the GTR+F+R4 model was chosen. Numbers at the nodes represent bootstrap support (BS) (%). MrBayes v3.2.6 [49] was used to construct the BI tree. The BI tree underwent 100,000 generations, and trees were sampled every 1000 generations, each with four independent Markov Chain Monte Carlo (MCMC). The best-fit substitution model was GTR+I+G. The first 25% sampled trees were discarded as burn-in. The remaining trees generated the consensus tree. Numbers at the nodes represent posterior probability (PP). The phylogenetic tree was visualized in iTOL v7 [50] and further edited in Adobe Illustrator 2021 for presentation.

## 3. Results

### 3.1. Morphological Characteristics of T. xanthipes

**Figure 1:**
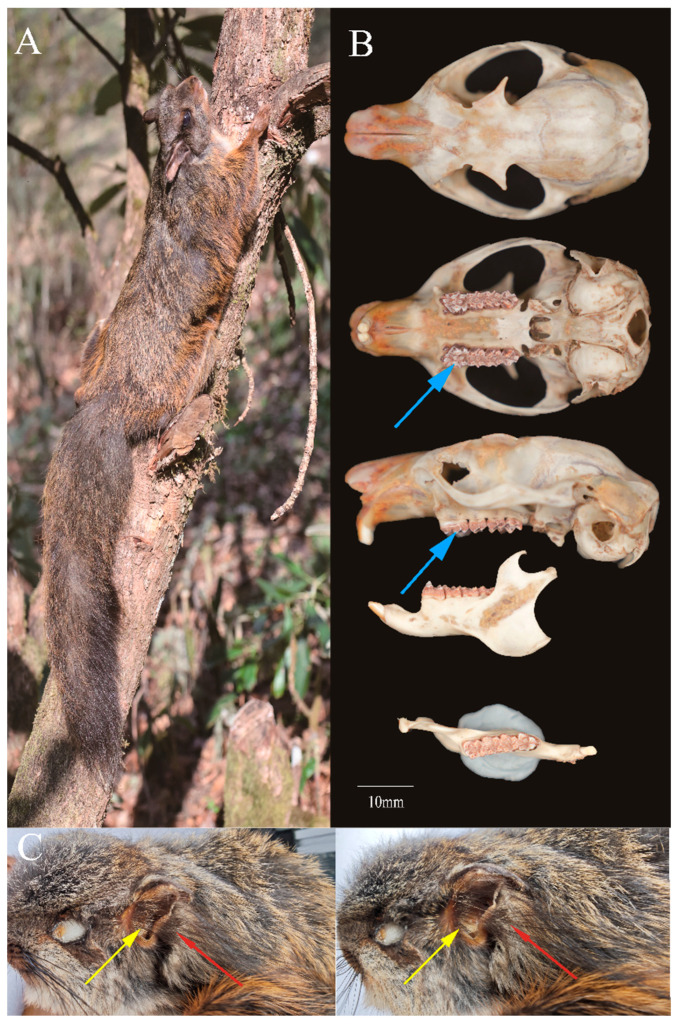
Morphology (**A**), cranium and mandible (**B**) and long black hair tufts on the inner and outer sides of base of the ears (**C**) of *Trogopterus xanthipes*. The blue arrow indicates the ridge on the crown of the cheekteeth, the yellow arrow indicates the inner tufts, and the red arrow indicates the outer tufts. It is the picture of the voucher specimen, whose genome was sequenced.

In this study, we collected one male *T. xanthipes* specimen. *Trogopterus xanthipes* is distinguished from other squirrels by tufts of long black hair on the inner and outer sides of base of the ears (Figure 1C) and numerous ridges on the crowns of the upper and lower cheekteeth (Figure 1B). *Trogopterus xanthipes* has a medium-sized body relative to other Pteromyini: head–body length (290 mm), tail length (270 mm), hindfoot length (55 mm), and ear length (30 mm). The dorsal pelage is gray at the base and brown to reddish at the tips; overall, it appears uniform reddish brown on dorsum and similar, lighter, and browner on venter.

The tail is slightly shorter than the head–body length. The distal end of the tail has scattered black long hairs. The cranium has a teardrop shape, with the rostrum narrower than the braincase and widest at the zygomatic bone.

### 3.2. Mitogenome Organization of T. xanthipes

**Figure 2:**
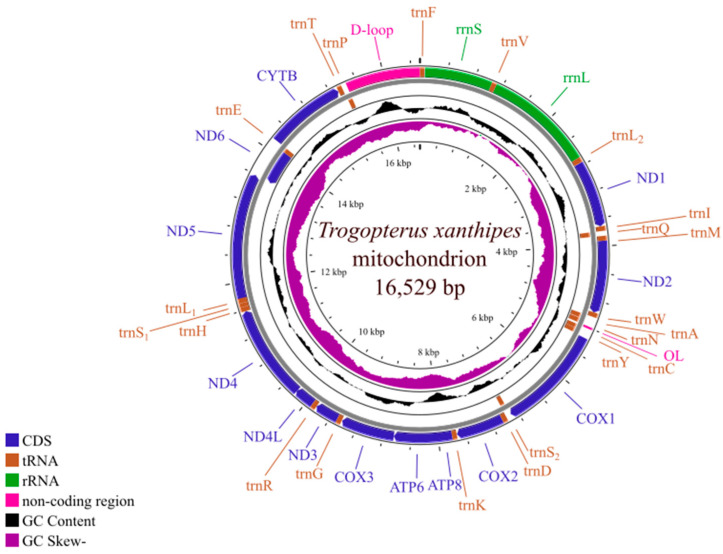
The circular map of the *Trogopterus xanthipes* mitogenome.

The *T. xanthipes* mitogenome is a closed double-stranded circular DNA molecule of 16,529 bp and contains the complete set of 37 genes (13 protein-coding genes, 2 rRNA genes, 22 tRNA genes) that are usually found in metazoans. (Figure 2). The *T. xanthipes* mitogenome has seven overlapping regions ranging from 1 to 43 bp in size, the largest overlapping region being between *ATP8* and *ATP6*. There were 18 intergenic spacer regions ranging from 1 bp to 8 bp in size, the largest intergenic spacer was 8 bp between *trnY* and *COX1* (Table S2). Nucleotide composition of the mitogenome was A (32.4%), T (31.5%), C (23.8%), G (12.4%), and AT content (63.9%) was higher than GC content (36.1%) of *T. xanthipes* mitogenome. AT-skew and GC-skew of *T. xanthipes* mitogenome were 0.015 and −0.316, respectively (Table S3).

### 3.3. Protein-Coding Genes and Codon Usage Patterns

**Figure 3:**
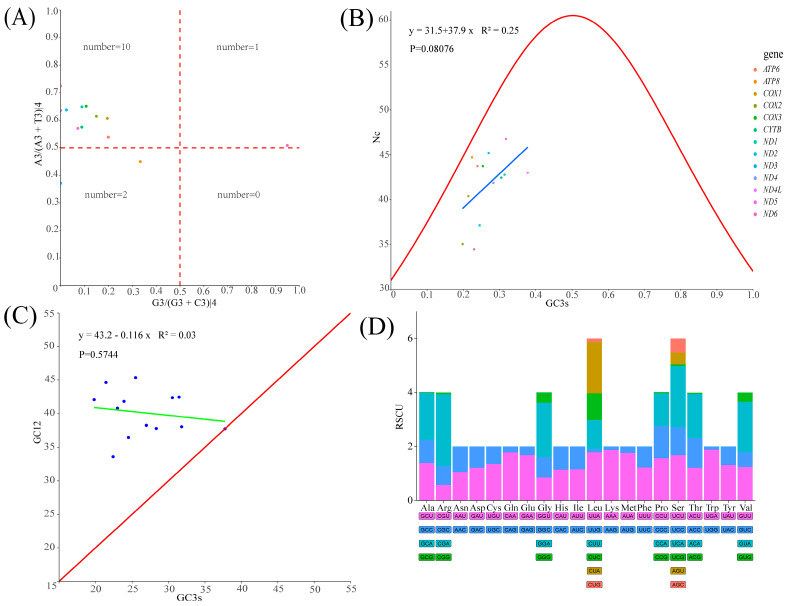
Codon usage preference analysis of the *Trogopterus xanthipes* mitogenome. (**A**) PR2, (**B**) ENC-plot (The linear expression y = 31.5 + 37.9x is shown as a blue line in the figure), (**C**) Neutral curve (the linear expression y = 43.2 − 0.116x is shown as a green line in the figure, blue dots represent protein-coding genes, (GC3_S_: GC content of the third position of synonymous codon; GC12: average value of the first and second positions of synonymous codon; Nc: effective number of codons; R^2^ represents the correlation, P represents the significant difference)), (**D**) RSCU.

**Figure 4:**
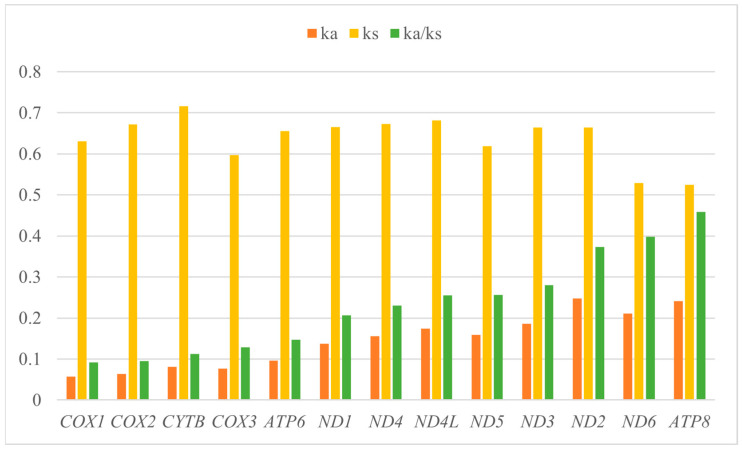
Evolutionary mutation rates (Ka/Ks) of 13 protein-coding genes of the *Trogopterus xanthipes* mitogenome.

The total length of 13 protein-coding genes (PCG) was 11,400 bp, accounting for 69% of *T. xanthipes* mitogenome (16,529 bp). The average AT content of 13 PCGs was about 64%. The 13 protein-coding genes use the typical ATN as the start codon, but there are various types of the stop codons: the stop codon of ND2 was TAG; the stop codon of ND6 and CYTB were AGA; COX3, ND3 and ND4 have incomplete TA-/T-- as the stop codon; the remaining seven protein-coding genes used the typical TAA as the stop codon. We used PR2, ENC-plot, Neutral curve, and RSCU to analyze the reasons that affected codon usage preference. PR2 showed that no protein-coding genes were located in the central site (0.5, 0.5), ND6 had A/G preference, ND3 and ATP8 had T/C preference, and the remaining protein-coding genes had A/C preference (Figure 3A). The ENC-plot showed that all protein-coding genes were located below the standard curve, indicating that the codon usage preference of all protein-coding genes was influenced by natural selection (Figure 3B). The Neutral curve showed that GC12 had no significant linear relationship with GC3s of 13 protein-coding genes (Figure 3C). The RSCU of 13 protein-coding genes was analyzed and found that 30 codons were preference codons (RSCU > 1), most of which ended in A/T(U) (Figure 3D), AUU, CUA, UUA, and AUA were the most frequently used (Table S4). Ka and Ks of 13 protein-coding genes were calculated. The Ka/Ks ratio of 13 PCGs was less than 1. The lowest rates of 13 PCGs were: *COX1* < *COX2* < *CYTB* < *COX3* < *ATP6* < *ND1 < ND4* < *ND4L* < *ND5* < *ND3* < *ND2* < *ND6* < *ATP8* (Figure 4). K2P genetic distances among 13 PCGs in the *T. xanthipes* mitogenome were calculated, indicating that the *COX1* gene had the smallest distance, and the *ND2* gene had the largest distance (Figure S1). Nucleotide diversity ranged between 0.177 and 0.715. The result showed that the *COX1* gene had the lowest nucleotide diversity, and the *ND1* gene had the highest nucleotide diversity (Figure S2).

### 3.4. Ribosomal RNA and Transfer RNA Genes

Both *rrnS* (969 bp in size) and *rrnL* (1572 bp in size) were encoded on the H-strand and were separated by *trnV* gene. The AT content of *rrnS* was 61%. The AT-skew and GC-skew were 0.157 and −0.127, respectively. The AT content of *rrnL* was 64.9%, and the AT-skew and GC-skew were 0.146 and −0.063, respectively (Table S3). Except for *trnS_1_*, which lacks the D-arm (DHU), the remaining 21 tRNAs have typical cloverleaf secondary structure (Figure S3). The length of 22 tRNAs ranged from 59 to 75 bp, of which the *trnS_1_* gene was the shortest (59 bp in size), and *trnL_2_* gene was the longest (75 bp in size). The secondary structure of 22 tRNAs have 39 pairs base mismatches: 31 G-U mismatches, 3 U-U mismatches, 2 A-A mismatches, and 2 A-C mismatches (Figure S3). There were base mismatches in 19 tRNA genes, most of which were G-U weak mismatch.

### 3.5. Non-Coding Region

The *T. xanthipes* mitogenome has two non-coding regions, L-strand replication origin (OL) and D-loop region. OL is located in the WANCY region, which contains 5 tRNA genes (*trnW*, *trnA*, *trnN*, *trnC*, *trnY*). OL (31bp in size) can be folded into the stable stem-loop secondary structure, the stem contained 7 G-C base pairs and enriched T bases in the loop (Figure S3). The D-loop region is the main non-coding region, and was located between *trnP* and *trnF* genes. The AT content in the D-loop region was 63.7%, and the AT-skew and GC-skew were −0.037 and −0.373, respectively (Table S3).

### 3.6. Phylogenetic Analysis

**Figure 5:**
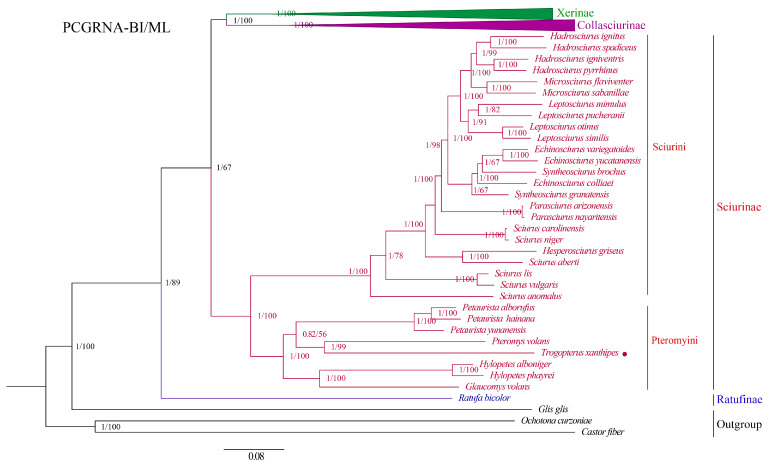
Maximum likelihood (ML) and Bayesian inference (BI) mitogenomic phylogenetic tree of Sciuridae (94 species) based on 13 protein-coding genes and 2 rRNA genes (PCGRNA). Node labels show the posterior probability (PP)/bootstrap support (BS) (%) values. Four Sciuridae subfamilies are marked by different colors. *Trogopterus xanthipes* is marked by a red dot.

We obtained four phylogenetic trees from two concatenated data matrices (PCGRNA and PCG12RNA) and two methods (ML and BI) (Figure 5 and Figure S4). Both methods yielded the same topologies with high node support values. The phylogenetic tree supported that Ratufinae, Sciurinae, Xerinae, and Callosciurinae formed four clades and were monophyletic with high support value. The phylogenetic relationships among subfamilies were ((Xerinae + Callosciurinae) + Sciurinae) + Ratufinae. Node support values of the BI tree were slightly higher than node support values of the ML tree. The Callosciurinae was the sister taxon of the Xerinae with strong support value (PP = 1, BS = 100%). All phylogenetic trees shared the same topology with little difference (Figure 5 and Figure S4) and supported monophyletic lineage of each genus of Sciuridae. *Trogopterus xanthipes* was sister species to *Pteromys volans* (Linnaeus, 1758) with strong support value (PP = 1, BS = 99%). The relationship within the Sciurinae clade was ((*Glaucomys* + *Hylopetes*) + ((*Trogopterus*+*Pteromys*) + *Petaurista*) + *Sciurus*). The relationships within the Callosciurinae clade was *Exilisciurus* + ((*Tamiops* + *Dremomys*) + ((*Lariscus* + *Sundasciurus*) + *Callosciurus*)). The relationship within the Xerinae clade was *Sciurotamias* + (*Tamias* + (*Callospermophilus* + (*Marmota* + (*Spermophi lus* + (*Urocitellus* + (*Ictidomys* + *Cynomys*)))))). Species within the same genus formed different minor clades. The tribe Pteromyini was the sister taxon of the tribe Sciurini with a strong support value (PP = 1, BS = 100%). The tribe Pteromyini was not in a separate clade and clustered with the tribe Sciurini.

## 4. Discussion

### 4.1. Morphological Characteristics and Mitogenome of T. xanthipes

Morphological characteristics are the basis for systematic taxonomy. Accurate species identification based on morphological characteristics is very important. *Trogopterus xanthipes* is a medium-sized species in Pteromyini. Its common name refers to the numerous ridges on the crowns of the upper and lower cheekteeth. The *T. xanthipes* mitogenome was reported for the first time in this study. The sequence is 16,529 bp in size and contains 37 genes and 2 non-coding regions. Like most squirrels, the *T. xanthipes* mitogenome was conserved both in terms of number and order of genes [51,52], neither gene rearrangement nor fragmentation were detected. The AT content of the *T. xanthipes* mitogenome was higher (63.9%) than the GC content (36.1%), and this was also observed in the mitogenomes of other species within Sciuridae [53,54]. The vAT-skew of 0.015 and GC-skew of −0.316 were detected in the *T. xanthipes* mitogenome. AT-skew is positive and GC-skew is negative in most metazoans [55], one possible explanation for the bias in base composition is the occurrence of asymmetric mutations during replication and transcription, as the subsequent selective pressure acting on these mutations [29].

The 13 protein-coding genes used the typical ATN as the start codon, but there were various types of the stop codons: the stop codon of *ND2* was TAG; the stop codons of *ND6* and *CYTB* were AGA; *COX3*, *ND3,* and *ND4* have incomplete TA-/T-- as the stop codon. The remaining seven protein-coding genes used the typical TAA as the stop codon. Usually, incomplete stop codons are transcribed and modified by polyadenylation forming complete stop codons [56]. We used PR2 (Figure 3A), ENC-plot (Figure 3B), Neutral curve (Figure 3C), and RSCU (Figure 3D) to analyze the reasons that affected codon usage preference. PR2 analysis revealed that no gene was located at coordinates (0.5, 0.5). It indicated that the genes been predominantly shaped by natural selection during evolution. ENC-plot analysis showed that all protein-coding genes of *T. xanthipes* were located below the standard curve, indicating that the codon usage preference of all the protein-coding genes was influenced by natural selection. The Nc values of 13PCGs ranged from 34.45 to 46.75, the Nc of *ND6* being less than 35, which indicated a significant preference for codon usage [57]. At the same time, the Nc value was also an essential index for evaluating the expression of endogenous genes. The codon preference of highly expressed genes was greater, and their Nc values were smaller. On the other hand, under-expressed genes contain a greater variety of rare codons and have larger Nc values [58]. The Neutral curve showed that genes GC12 and GC3s of 13 PCGs have no significant linear relationship, and the genes were scattered, indicating that these genes were mainly affected by selection during evolution. In summary, it can be deduced that the main reason for codon preference of 13 PCGs in *T. xanthipes* was selection pressure. We analyzed the relative synonymous codon usage (RSCU) of 13 protein-coding genes and evaluated the synonymous codon usage bias. RSCU > 1.6 indicates overrepresented codon, while RSCU > 1 indicated strong preference for that codon. RSCU of 1 indicated no preference, meaning all codons were used equally, and RSCU < 1 indicated weak preference [59]. It was noted that the high-frequency codons predominantly end in A/U, while the codons ending with G/C were either rarely used or not used at all.

Ka/Ks < 1 of 13 PCGs in *T. xanthipes* mitogenome indicated that 13 PCGs were mainly subject to purifying selection to maintain gene functional conservation during evolution. *COX1* gene had the slowest evolutionary mutation rate, *ATP8* gene had the fastest evolutionary mutation rate, which is a general feature of the tribe Pteromyini [60]. K2P distances analysis indicated that the *COX1* gene had the smallest distance, and the *ND2* gene had the largest distance. Nucleotide diversity analysis of this *T. xanthipes* showed that the *COX1* gene had the lowest nucleotide diversity, and the *ND1* gene had the highest nucleotide diversity among all mitochondrial genes in *T. xanthipes*. The nucleotide sequence of the *COX1* gene is commonly used as a taxon barcode and molecular marker [61], however, it is highly conserved in *T. xanthipes*. The marked decrease in genetic variation in monotypic populations likely results from minimal genetic diversity in small populations, impeding gene flow and amplifying genetic drift. We presumed that low genetic variation of *Trogopterus* may stem from its small population size and restricted range. Species with higher genetic diversity are more adaptable to environmental shifts and can expand their ranges more readily, while those with lower diversity are less resilient, less adaptable, and more vulnerable to extinction.

There were 39 pairs base mismatches in 22 tRNA genes, from which G-U mismatch (31 pairs) was the most common, mainly occurring at the intersection of DHU arm, TΨC arm, acceptor arm, and anticodon arm. It is important to maintain the stability of the tRNA secondary structure [62]. Transfer RNAs (tRNAs) usually possess typical cloverleaf secondary structures, except for loss of the dihydrouracil arm of *trnS_1_* (which is a common feature of the mitogenome of metazoans [55]) in the *T. xanthipes* mitogenome. It is noteworthy that *trnK* of *Apodemus draco* (Barrett-Hamilton, 1900) (Rodentia: Muridae) failed to form a typical cloverleaf secondary structure due to lacking the DHU loop [63]. More studies are required to confirm structure variation of tRNAs in different taxa in the future.

The D-loop region does not encode genes and has relatively small selection pressure in the evolutionary process, so the evolutionary mutation rate is fast and the degree of sequence variation is high, producing the differences in the sequence between different species. The D-loop region sequence has been proven to be an effective molecular marker for genetic differentiation studies of closely related populations [52,64]. Tandem repeat sequences were found in the D-loop region of most mammals [65], but not in the *T. xanthipes* mitogenome, although it is yet to be determined whether this is a general feature of the tribe Pteromyini worth further studying. OL region (31 bp in size) is a small non-coding region in *T. xanthipes* mitogenome. OL was located in the WANCY regions (*trnW*, *trnA*, *trnN*, *trnC*, *trnY*) and is prevalent in mammals [66].

### 4.2. Comparative Mitogenomic Analysis of T. xanthipes and the Closest Related Species (P. volans)

The mitogenomes of *T. xanthipes* and *P. volans* are highly conserved structures that usually consist of a single circular molecule, and contain 37 genes. Both species exhibited AT bias in nucleotide composition, with AT contents of 63.9% (*T. xanthipes*) and 62.6% (*P. volans*). All tRNAs had the typical cloverleaf structure except for *trnS_1_* (without the dihydrouridine (DHU) arm). In most mammalian species, the D-loop region serves as a regulatory region for mitochondrial DNA expression and replication, with varying lengths [52]. The D-loop region of *T. xanthipes* (1077 bp) is longer than that of *P. volans* (1066 bp). There were uniform start codons for protein-coding genes of *T. xanthipes* and *P. volans*. However, differences in stop codons for protein-coding genes of *P. volans*: *AGG* was the stop codon of *ND6* gene; incomplete stop codons (T--) was used for *ND2*. *Pteromys volans* shows the slowest evolutionary mutation rate in the *COX2* gene. PR2 showed similar preference for protein-coding genes of *T. xanthipes* and *P. volans,* except the *ND3* gene had A/C preference. Both species had similar results in ENC-plot, Neutral curve, and RSCU analyses.

### 4.3. Phylogeny of the Sciuridae Based on Mitogenomes Sequences

Phylogenetic analysis of mitogenomic sequences can provide valuable insights into the evolutionary relationships among different species and groups of animals. We obtained two trees from two concatenated data matrices (PCGRNA: protein-coding and rRNA gene sequences; PCG12RNA: same as PCGRNA but the third codon sequences of protein-coding genes excluded; the third codon positions may suffer from mutation saturation which can bring noise to the phylogenetic analysis) based on two tree-construction methods (ML and BI). The two trees have similar topologies to each other and consistently support the monophyly of the family Sciuridae in different datasets (PCGRNA and PCG12RNA). Sciuridae includes the following subfamilies: Ratufinae, Sciurinae, Xerinae, Callosciurinae, and Sciurillinae. In the current study, we reconstructed the phylogeny of the family Sciuridae. Due to the absence of the Sciurillinae mitogenomes, this subfamily was not included in the phylogenetic analyses. The phylogenetic trees supported that Ratufinae, Sciurinae, Xerinae, and Callosciurinae formed four clades and were monophyletic with strong support values. The phylogenetic relationship among subfamilies was ((Xerinae + Callosciurinae) + Sciurinae) + Ratufinae. Both phylogenetic methods produced the same tree topology and supported monophyly of each genus of Sciuridae. The Callosciurinae was the sister taxon of the Xerinae with strong support value. The phylogenetic arrangement described above agrees with previous Sciuridae phylogenies based on nuclear genes (Steppan et al., 2004) [12] and complete mitochondrial genomes (Cong et al., 2016) [67]. Our phylogeny contradicts the result of Mercer and Roth (2003) [11] who treated Xerinae and Sciurinae as sister groups. The Ratufinae placed the root of phylogenetic trees in this study, which supported previous studies [11,67]. Node support values of BI tree was slightly higher than node support values of the ML tree. Four phylogenetic trees shared the same topology with little difference in support value (Figure 5 and Figure S4). *Trogopterus xanthipes* was sister species to *P. volans* with a strong support value (PP = 1, BS = 99%), which is consistent with the result of Lu et al. (2012) [68] based on Bayesian DIVA. The *Petaurista hainana* (Allen, 1925) was closely related to the *Petaurista alborufus* (Milne-Edwards, 1870) in the *Petaurista*, and then it clustered with *Petaurista yunanensis* (Anderson, 1875), which is consistent with result of Yu et al. (2006) [69] and Li et al. (2020) [70]. *Marmota*, *Spermophilus*, *Urocitellus*, *Ictidomys*, and *Cynomys* in the Xerinae first clustered into one branch, next clustered with *Callospermophilus*, and then clustered with *Tamias,* and finally clustered into *Sciurotamias*, which is consistent with the results of Zelditch et al. (2015) [71]. Previous studies in Callosciurinae have shown that *Dremomys* and *Tamias* first clustered into a single branch, and then clustered into one branch with *Callosciurus* and finally clustered into *Exilisciurus* [67,72,73], which was consistent with the results of this study. Species within the same genus form different minor clades, suggesting relatively high interspecific divergences. The tribe Pteromyini was the sister taxon of the tribe Sciurini with a strong support value (PP = 1, BS = 100%). The tribe Pteromyini was not in a separate clade and clustered with the tribe Sciurini which did not support the traditional division of Sciuridae into subfamily Petauristinae and Sciurinae. Our results support that Sciuridae division into four subfamilies: Ratufinae, Sciurinae, Xerinae, and Callosciurinae, except for Sciurillinae (data deficient). Our study obtained the mitogenome data of the monotypic genus *Trogopterus*. Compared with the previous studies [28,29], more mitogenome data of Sciuridae were included, which showed the phylogenetic relationship of Sciuridae to the greatest extent, and laid a foundation for the subsequent study of the genetic relationship and species evolution of Sciuridae.

## 5. Conclusions

Here, for the first time, we sequenced the *T. xanthipes* mitogenome and analyzed structural features and variation. No tandem repeats occurred in the D-loop of *T. xanthipes* mitogenome and there was a loss of the dihydrouracil arm of *trnS_1_*. Furthermore, we explored codon usage patterns, evolutionary mutation rates, K2P distances, and nucleotide diversity of different protein-coding genes. The results showed that the *COX1* gene was highly conserved. All phylogenetic trees shared same topology with little difference in support value and robustly supported the monophyly of the Sciuridae, with each subfamily forming a monophyletic clade. The phylogenetic relationship among the subfamilies was as follows: ((Xerinae + Callosciurinae) + Sciurinae) + Ratufinae, and supported monophyly of each genus of Sciuridae. *Trogopterus xanthipes* was sister species to *P. volans* with strong support value. The tribe Pteromyini was the sister taxon of the tribe Sciurini with strong support value (PP = 1, BS = 100%). Species within the same genus formed different minor clades. This study provides new insights into the evolutionary history and taxonomy of the Sciuridae. The *T. xanthipes* mitogenome provides novel molecular markers for studying the taxonomy and phylogeny of Sciuridae.

## Data Availability

The experimental data used to support the findings of this study are available from the corresponding authors on request.

## Supplementary Materials

The following supporting information can be downloaded at: https://www.mdpi.com/article/10.3390/ani15101493/s1, Figure S1: Kimura 2-parameter (K2P) genetic distances of 13 protein-coding genes from *Trogopterus xanthipes*; Figure S2: Nucleotide diversity (Pi) of the *Trogopterus xanthipes;* Figure S3: Secondary structure of 22 tRNA genes and secondary structure of L-strand replication origin (OL) region (31 bp) in the mitogenome of the *Trogopterus xanthipes* mitogenome (base mismatches are shown in red. Seven GC base pairs in the stem are shown in red, the enrichment with T-base in the loop is shown in green); Figure S4: Maximum likelihood (ML) and Bayesian inference (BI) mitogenomic phylogenetic tree of Sciuridae (94 species) based on first and second codon positions of the 13 PCGs and 2 rRNAs (PCG12RNA); Figure S5: Codon usage preference analysis of the *Pteromys volans* mitogenome. (**A**) PR2, (**B**) ENC-plot, (**C**) Neutral curve, (**D**) RSCU. Figure S6: Evolutionary mutation rates (Ka/Ks) of 13 protein-coding genes of the *Pteromys volans* mitogenome. Table S1: Species information; Table S2: Organization of the *Trogopterus xanthipes* mitogenome, Table S3: Nucleotide composition (%) and skewness of the *Trogopterus xanthipes* mitogenome, Table S4: The relative synonymous codon usage (RSCU) of the *Trogopterus xanthipes.* Table S5: Organization of the *Pteromys volans* mitogenome, Table S6: Nucleotide composition (%) and skewness of the *Pteromys volans* mitogenome, Table S7: The relative synonymous codon usage (RSCU) of the *Pteromys volans*.
