# Supplementary material for: Mitogenome of Endemic Species of Flying Squirrel, Trogopterus xanthipes (Rodentia, Mammalia) and Phylogeny of the Sciuridae
Source: Animals (Basel). 2025 May 21;15(10):1493. doi: 10.3390/ani15101493 (PMC12108527; doi:10.3390/ani15101493)
Supplement: Supplementary file 1 [file animals-15-01493-s001.zip › Table S1.pdf]

Table S1. Species information.

	Family	Subfamily	Genus	Species	GenBank number	Country
Outgroup	Gliridae	Glirinae	Glis	Glis glis	NC001892	Italy
	Castoridae		Castor	Castor fiber	NC028625	Germany
	Ochotonidae		Ochotona	Ochotona curzoniae	NC011029	China
Ingroup	Sciuridae	Ratufinae	Ratufa	Ratufa bicolor	NC023780	China
		Sciurinae	Sciurus	Parasciurus arizonensis	NC050011	Brazil
				Parasciurus nayaritensis	NC050014	Brazil
				Echinosciurus colliaei	NC050013	Brazil
				Echinosciurus variegatoides	NC050015	Brazil
				Echinosciurus yucatanensis	NC050016	Brazil
				Leptosciurus pucheranii	NC050017	Brazil
				Leptosciurus mimulus	NC050020	Brazil
				Leptosciurus otinus	NC050021	Brazil
				Leptosciurus similis	NC050022	Brazil
				Hadrosciurus igniventris	NC050027	USA
				Hadrosciurus pyrrhinus	NC050028	USA
				Hadrosciurus spadiceus	NC050029	USA
				Hadrosciurus ignitus	NC050032	Brazil
				Hesperosciurus griseus	NC050034	Brazil
				Microsciurus flaviventer	NC050030	Brazil
				Microsciurus sabanillae	NC050031	Brazil
				Sciurus aberti	MT211954	Brazil

				Sciurus lis	MT134013	Brazil
				Sciurus vulgaris	NC002369	Italy
				Sciurus carolinensis	NC050012	Brazil
				Sciurus anomalus	ON620284	Turkey
				Sciurus niger	KY411003	USA
				Syntheosciurus granatensis	NC050018	Brazil
				Syntheosciurus brochus	MT240890	Brazil
			Pteromys	Pteromys volans	NC019612	South Korea
			Hylopetes	Hylopetes alboniger	NC031847	China
				Hylopetes phayrei	NC026443	China
			Petaurista	Petaurista alborufus	MN011572	China
				Petaurista hainana	NC023089	China
				Petaurista yunanensis	NC033902	China
			Glaucomys	Glaucomys volans	NC050026	USA
		Callosciurinae	Callosciurus	Callosciurus erythraeus	KP708709	China
				Callosciurus finlaysonii	NC035817	German
				Callosciurus prevostii	NC035816	German
				Callosciurus pygerythrus	KY410913	India
				Callosciurus albescens	KY410906	Indonesia
				Callosciurus melanogaster	KY410908	Indonesia
				Callosciurus nigrovittatus	KY410866	Indonesia
				Callosciurus baluensis	KY410847	Malaysia
				Callosciurus orestes	KY410869	Malaysia
				Callosciurus adamsi	NC030071	Malaysia
				Callosciurus notatus	MT231329	Malaysia

				Callosciurus quinquestriatus	KY410891	Myanmar
				Callosciurus phayrei	KY410851	Myanmar
				Callosciurus caniceps	KY410858	Thailand
			Dremomys	Dremomys rufigenis	NC026442	China
				Dremomys pernyi	NC035577	China
				Dremomys pyrrhomerus	KP708705	China
				Dremomys lokriah	KY410879	India
				Dremomys gularis	OQ160785	Vietnam
			Tamiops	Tamiops swinhoei	NC026875	China
				Tamiops maritimus	NC029325	China
				Tamiops mcclellandii	NC080357	Vietnam
				Tamiops rodolphii	KY410983	Vietnam
			Exilisciurus	Exilisciurus exilis	NC030072	USA
			Lariscus	Lariscus insignis	NC030070	USA
			Sundasciurus	Sundasciurus brookei	NC035812	USA
				Sundasciurus altitudinis	MT157213	Indonesia
				Sundasciurus robinsoni	MT157214	Malaysia
				Sundasciurus lowii	MT157215	Malaysia
				Sundasciurus hippurus	MT157216	Malaysia
		Xerinae	Marmota	Marmota marmota marmota	MN935776	Austria
				Marmota monax	JAMOFY010003380	Canada
				Marmota flaviventris	NC042243	Canada
				Marmota himalayana	NC018367	China
				Marmota vancouverensis	NC048490	China
				Marmota baibacina	NC086592	Mongolia

				Marmota sibirica	NC086593	Mongolia
			Spermophilus	Spermophilus alashanicus	NC071768	China
				Spermophilus citellus	PQ533849	Turkey
				Spermophilus dauricus	KP708706	China
				Spermophilus taurensis	OQ675160	Turkey
				Spermophilus xanthoprymnus	PQ533851	Turkey
			Sciurotamias	Sciurotamias forresti	PP175169	China
				Sciurotamias davidianus	KP708708	China
			Tamias	Tamias sibiricus	NC025277	South Korea
				Tamias quadrivittatus	NC032370	USA
				Tamias rufus	NC032371	USA
				Tamias canipes	NC032372	USA
				Tamias dorsalis	NC032373	USA
				Tamias cinereicollis	NC032374	USA
				Tamias striatus	NC032375	USA
				Tamias umbrinus	NC032376	USA
				Tamias ruficaudus simulans	KY070177	USA
				Tamias amoenus luteiventris	KY070157	USA
			Callospermophilus	Callospermophilus lateralis	NC031210	China
			Cynomys	Cynomys ludovicianus	KP326310	China
				Cynomys leucurus	KP326309	China
				Cynomys gunnisoni gunnisoni	MG450794	USA
			Ictidomys	Ictidomys tridecemlineatus	KP698974	China
			Urocitellus	Urocitellus richardsonii	NC031209	China
				Urocitellus undulatus	PQ720778	China
