# Supplementary material for: Mitogenome of Endemic Species of Flying Squirrel, Trogopterus xanthipes (Rodentia, Mammalia) and Phylogeny of the Sciuridae
Source: Animals (Basel). 2025 May 21;15(10):1493. doi: 10.3390/ani15101493 (PMC12108527; doi:10.3390/ani15101493)
Supplement: Supplementary file 1 [file animals-15-01493-s001.zip › Table S2.pdf]

Table S2. Organization of the *Trogopterus xanthipes* mitogenome.

Gene	Position	Size(bp)	Intergenic nucleotides	Codon		Strand	Anticodon
				Start	Stop		
trnF	1-65	65				H	GAA
rrnS	72-1,040	969	6			H	
trnV	1,041-1,109	69				H	UAC
rrnL	1,110-2,681	1,572				H	
trnL$_2$	2,682-2,756	75				H	UAA
ND1	2,760-3,716	957	3	ATG	TAA	H	
trnI	3,717-3,785	69				H	GAU
trnQ	3,783-3,854	72	-3			L	UUG
trnM	3,857-3,924	68	2			H	CAU
ND2	3,926-4,969	1,044	1	ATC	TAG	H	
trnW	4,968-5,035	68	-2			H	UCA
trnA	5,039-5,107	69	3			L	UGC
trnN	5,114-5,186	73	6			L	GUU
OL	5,187-5,217	31				L	
trnC	5,218-5,284	67				L	GCA
trnY	5,286-5,351	66	1			L	GUA
COX1	5,360-6,901	1,542	8	ATG	TAA	H	
trnS$_2$	6,905-6,973	69	3			L	UGA
trnD	6,977-7,045	69	3			H	GUC
COX2	7,047-7,730	684	1	ATG	TAA	H	
trnK	7,734-7,801	68	3			H	UUU
ATP8	7,803-8,006	204	1	ATG	TAA	H	
ATP6	7,964-8,644	681	-43	ATG	TAA	H	
COX3	8,644-9,423	780	-1	ATG	T--	H	
trnG	9,428-9,496	69	4			H	UCC
ND3	9,497-9,843	347		ATA	TA-	H	
trnR	9,844-9,911	68				H	UCG
ND4L	9,913-10,209	297	1	ATG	TAA	H	
ND4	10,203-11,580	1,378	-7	ATG	T--	H	
trnH	11,581-11,648	68				H	GUG
trnS$_1$	11,649-11,707	59				H	GCU
trnL$_1$	11,708-11,777	70				H	UAG
ND5	11,769-13,592	1,824	-9	ATA	TAA	H	
ND6	13,576-14,100	525	-17	ATG	AGA	L	
trnE	14,101-14,169	69				L	UUC
CYTB	14,171-15,313	1,143	1	ATA	AGA	H	
trnT	15,316-15,383	68	2			H	UGU
trnP	15,389-15,457	69	5			L	UGG
D-loop	15,458-16,529	1,072				H	
