# Supplementary material for: Mitogenome of Endemic Species of Flying Squirrel, Trogopterus xanthipes (Rodentia, Mammalia) and Phylogeny of the Sciuridae
Source: Animals (Basel). 2025 May 21;15(10):1493. doi: 10.3390/ani15101493 (PMC12108527; doi:10.3390/ani15101493)
Supplement: Supplementary file 1 [file animals-15-01493-s001.zip › Table S3.pdf]

Table S3. Nucleotide composition (%) and skewness of the *Trogopterus xanthipes* mitogenome.

Regions	Size (bp)	T(U)	C	A	G	AT (%)	AT-skew	GC-skew
ATP6	681	33.8	25.4	30.5	10.3	64.3	-0.05	-0.424
ATP8	204	32.8	25	37.7	4.4	70.5	0.069	-0.7
COX1	1,542	34.2	20.8	29	16.1	63.2	-0.082	-0.127
COX2	684	31.9	21.6	33.6	12.9	65.5	0.027	-0.254
COX3	779	31.8	24.4	29.3	14.5	61.1	-0.042	-0.254
ND1	957	31.5	27.7	29.9	11	61.4	-0.026	-0.432
ND2	1,044	32.6	24.9	35	7.6	67.6	0.035	-0.534
ND3	347	37.5	23.3	28.2	11	65.7	-0.14	-0.361
ND4	1,378	33.4	25.5	32	9.1	65.4	-0.021	-0.476
ND4L	297	35	26.9	27.6	10.4	62.6	-0.118	-0.441
ND5	1,824	32.2	26	31.9	9.9	64.1	-0.004	-0.447
ND6	525	40.8	6.5	24.4	28.4	65.2	-0.251	0.628
CYTB	1,143	32.9	26.2	28.7	12.2	61.6	-0.068	-0.362
PCGs	11,400	33.3	23.9	30.7	12.1	64	-0.041	-0.33
rrnS	969	25.7	22	35.3	17	61	0.157	-0.127
rrnL	1,572	27.7	18.7	37.2	16.5	64.9	0.146	-0.063
rRNAs	2,541	26.9	20	36.4	16.7	63.3	0.15	-0.089
tRNAs	1,507	31.4	16.9	32.6	19.1	64	0.019	0.061
D-loop	1,072	33	24.9	30.7	11.4	63.7	-0.037	-0.373
Full sequence	16,529	31.5	23.8	32.4	12.4	63.9	0.015	-0.316
