# Supplementary material for: Mitogenome of Endemic Species of Flying Squirrel, Trogopterus xanthipes (Rodentia, Mammalia) and Phylogeny of the Sciuridae
Source: Animals (Basel). 2025 May 21;15(10):1493. doi: 10.3390/ani15101493 (PMC12108527; doi:10.3390/ani15101493)
Supplement: Supplementary file 1 [file animals-15-01493-s001.zip › Table S4.pdf]

Table S4. The relative synonymous codon usage (RSCU) of the *Trogopterus xanthipes*.

Amino acid	Codon	Count	RSCU	Amino acid	Codon	Count	RSCU
Ala	GCU	82	1.39	Asn	AAU	86	1.06
	GCC	50	0.85		AAC	77	0.94
	GCA	103	1.75	Pro	CCU	76	1.57
	GCG	1	0.02		CCC	58	1.2
Cys	UGU	17	1.36		CCA	58	1.2
	UGC	8	0.64		CCG	2	0.04
Asp	GAU	41	1.22	Gln	CAA	75	1.79
	GAC	26	0.78		CAG	9	0.21
Glu	GAA	84	1.68	Arg	CGU	9	0.58
	GAG	16	0.32		CGC	11	0.71
Phe	UUU	150	1.23		CGA	41	2.65
	UUC	94	0.77		CGG	1	0.06
Gly	GGU	46	0.86	Ser	UCU	84	1.68
	GGC	40	0.75		UCC	52	1.04
	GGA	107	2.01		UCA	113	2.26
	GGG	20	0.38		UCG	3	0.06
His	CAU	54	1.15		AGU	22	0.44
	CAC	40	0.85		AGC	26	0.52
Ile	AUU	215	1.16	Thr	ACU	92	1.22
	AUC	157	0.84		ACC	83	1.1
Lys	AAA	91	1.88		ACA	123	1.63
	AAG	6	0.12		ACG	3	0.04
Leu	UUA	184	1.79	Val	GUU	53	1.25
	UUG	12	0.12		GUC	23	0.54
	CUU	110	1.07		GUA	79	1.86
	CUC	102	0.99		GUG	15	0.35
	CUA	195	1.9	Trp	UGA	99	1.89
	CUG	13	0.13		UGG	6	0.11
Met	AUA	195	1.77	Tyr	UAU	84	1.32
	AUG	25	0.23		UAC	43	0.68
