# Supplementary material for: Mitogenome of Endemic Species of Flying Squirrel, Trogopterus xanthipes (Rodentia, Mammalia) and Phylogeny of the Sciuridae
Source: Animals (Basel). 2025 May 21;15(10):1493. doi: 10.3390/ani15101493 (PMC12108527; doi:10.3390/ani15101493)
Supplement: Supplementary file 1 [file animals-15-01493-s001.zip › Table S5.pdf]

Organization of the *Pteromys volans* mitogenome.

Gene	Position	Size	Intergenic nucleotides	Codon		Strand	Anticodon
				Start	Stop		
trnF	1-70	70				H	GAA
rrnS	71-1036	966				H	
trnV	1,037-1,104	68				H	UAC
rrnL	1,105-2,669	1,565				H	
trnL$_2$	2,671-2,745	75	1			H	UAA
ND1	2,749-3,705	957	3	ATG	TAA	H	
trnI	3,706-3,774	69				H	GAU
trnQ	3,772-3,843	72	-3			L	UUG
trnM	3,846-3,914	69	2			H	CAU
ND2	3,915-4,956	1,042		ATT	T	H	
trnW	4,957-5,023	67				H	UCA
trnA	5,026-5,094	69	2			L	UGC
trnN	5,104-5,176	73	9			L	GUU
trnC	5,207-5,273	67	31			L	GCA
trnY	5,275-5,340	66	1			L	GUA
COX1	5,349-6,890	1,542	8	ATG	TAA	H	
trnS$_2$	6,895-6,963	69	4			L	UGA
trnD	6,967-7,035	69	3			H	GUC
COX2	7,037-7,720	684	1	ATG	TAA	H	
trnK	7,724-7,792	69	3			H	UUU
ATP8	7794-7997	204	1	ATG	TAA	H	
ATP6	7,955-8,635	681	-43	ATG	TAA	H	
COX3	8,635-9,418	784	-1	ATG	T	H	
trnG	9,419-9,489	71				H	UCC
ND3	9,490-9,836	347		ATA	TA	H	
trnR	9,837-9,902	66				H	UCG
ND4L	9,904-10,200	297	1	ATG	TAA	H	
ND4	10,194-11,571	1,378	-7	ATG	T	H	
trnH	11,572-11,639	68				H	GUG
trnS$_1$	11,641-11,697	57	1			H	GCU
trnL$_1$	11,699-11,768	70	1			H	UAG
ND5	11,769-13,586	1,818		ATA	TAA	H	
ND6	13,570-14,094	525	-17	ATG	AGG	L	
trnE	14,095-14,163	69				L	UUC
CYTB	14,168-15,307	1,140	4	ATG	AGA	H	
trnT	15,308-15,375	68				H	UGU
trnP	15,379-15,447	69	3			L	UGG
D-loop	15,448-16,513	1,066				H	
