# Supplementary material for: Mitogenome of Endemic Species of Flying Squirrel, Trogopterus xanthipes (Rodentia, Mammalia) and Phylogeny of the Sciuridae
Source: Animals (Basel). 2025 May 21;15(10):1493. doi: 10.3390/ani15101493 (PMC12108527; doi:10.3390/ani15101493)
Supplement: Supplementary file 1 [file animals-15-01493-s001.zip › Table S6.pdf]

Nucleotide composition (%) and skewness of the *Pteromys volans* mitogenome.

Regions	Size (bp)	T(U)	C	A	G	AT (%)	AT-skew	GC-skew
ATP6	681	32.6	27.6	28.9	10.9	61.5	-0.06	-0.435
ATP8	204	33.3	24	34.8	7.8	68.1	0.022	-0.508
COX1	1,542	32.9	21.9	28.9	16.2	61.8	-0.065	-0.15
COX2	684	30.3	24.3	32.6	12.9	62.9	0.037	-0.307
COX3	784	31.1	26.1	27.9	14.8	59	-0.054	-0.277
CYTB	1,140	30.9	28.7	27.9	12.5	58.8	-0.051	-0.391
ND1	957	28.5	29.6	30.4	11.5	58.9	0.032	-0.44
ND2	1,042	31.3	26.7	34.2	7.9	65.5	0.044	-0.544
ND3	347	31.7	28.2	29.1	11	60.8	-0.043	-0.441
ND4	1,378	32.8	26.6	30.9	9.7	63.7	-0.03	-0.464
ND4L	297	37.4	24.2	27.6	10.8	65	-0.15	-0.385
ND5	1,818	30.1	27.5	31.7	10.7	61.8	0.027	-0.441
ND6	525	41.5	6.5	24.8	27.2	66.3	-0.253	0.616
PCGs	11,394	31.9	25.5	30.2	12.5	62.1	-0.028	-0.343
rrnL	1,565	27.5	18.8	37.4	16.2	64.9	0.152	-0.075
rrnS	966	25.6	21.8	35.7	16.9	61.3	0.166	-0.128
rRNAs	2,531	26.8	20	36.7	16.5	63.5	0.157	-0.096
tRNAs	1,510	31.8	16.5	33.7	18	65.5	0.029	0.044
D-loop	1,066	30.6	26.4	31.8	11.3	62.4	0.019	-0.401
Full genome	16,513	30.4	24.9	32.2	12.6	62.6	0.029	-0.328
