# Supplementary material for: Mitogenome of Endemic Species of Flying Squirrel, Trogopterus xanthipes (Rodentia, Mammalia) and Phylogeny of the Sciuridae
Source: Animals (Basel). 2025 May 21;15(10):1493. doi: 10.3390/ani15101493 (PMC12108527; doi:10.3390/ani15101493)
Supplement: Supplementary file 1 [file animals-15-01493-s001.zip › Table S7.pdf]

The relative synonymous codon usage (RSCU) of the *Pteromys volans*.

| Amino acid | Codon | Count | RSCU | Amino acid | Codon | Count | RSCU |
|---|---|---|---|---|---|---|---|
| Ala | GCU | 58 | 0.95 | Asn | AAU | 69 | 0.86 |
| | GCC | 84 | 1.37 | | AAC | 92 | 1.14 |
| | GCA | 102 | 1.67 | Pro | CCU | 60 | 1.27 |
| | GCG | 1 | 0.02 | | CCC | 78 | 1.65 |
| Cys | UGU | 10 | 0.74 | | CCA | 46 | 0.97 |
| | UGC | 17 | 1.26 | | CCG | 5 | 0.11 |
| Asp | GAU | 34 | 1 | Gln | CAA | 73 | 1.82 |
| | GAC | 34 | 1 | | CAG | 7 | 0.17 |
| Glu | GAA | 84 | 1.7 | Arg | CGU | 12 | 0.74 |
| | GAG | 15 | 0.3 | | CGC | 11 | 0.68 |
| Phe | UUU | 139 | 1.13 | | CGA | 39 | 2.4 |
| | UUC | 108 | 0.87 | | CGG | 3 | 0.18 |
| Gly | GGU | 30 | 0.57 | Ser | UCU | 70 | 1.39 |
| | GGC | 58 | 1.09 | | UCC | 70 | 1.39 |
| | GGA | 103 | 1.94 | | UCA | 106 | 2.11 |
| | GGG | 21 | 0.4 | | UCG | 6 | 0.12 |
| His | CAU | 40 | 0.84 | | AGU | 21 | 0.42 |
| | CAC | 55 | 1.16 | | AGC | 29 | 0.58 |
| Ile | AUU | 215 | 1.21 | Thr | ACU | 82 | 1.09 |
| | AUC | 140 | 0.79 | | ACC | 80 | 1.06 |
| Lys | AAA | 85 | 1.85 | | ACA | 129 | 1.71 |
| | AAG | 7 | 0.15 | | ACG | 10 | 0.13 |
| Leu | UUA | 164 | 1.6 | Val | GUU | 49 | 1.17 |
| | UUG | 13 | 0.13 | | GUC | 35 | 0.83 |
| | CUU | 114 | 1.11 | | GUA | 73 | 1.74 |
| | CUC | 94 | 0.92 | | GUG | 11 | 0.26 |
| | CUA | 210 | 2.05 | Trp | UGA | 97 | 1.87 |
| | CUG | 20 | 0.2 | | UGG | 7 | 0.13 |
| Met | AUA | 190 | 1.65 | Tyr | UAU | 68 | 1.02 |
| | AUG | 41 | 0.35 | | UAC | 65 | 0.98 |
