# Supplementary figures and images for: Mitogenome of Endemic Species of Flying Squirrel, Trogopterus xanthipes (Rodentia, Mammalia) and Phylogeny of the Sciuridae
Source: Animals (Basel). 2025 May 21;15(10):1493. doi: 10.3390/ani15101493 (PMC12108527; doi:10.3390/ani15101493)

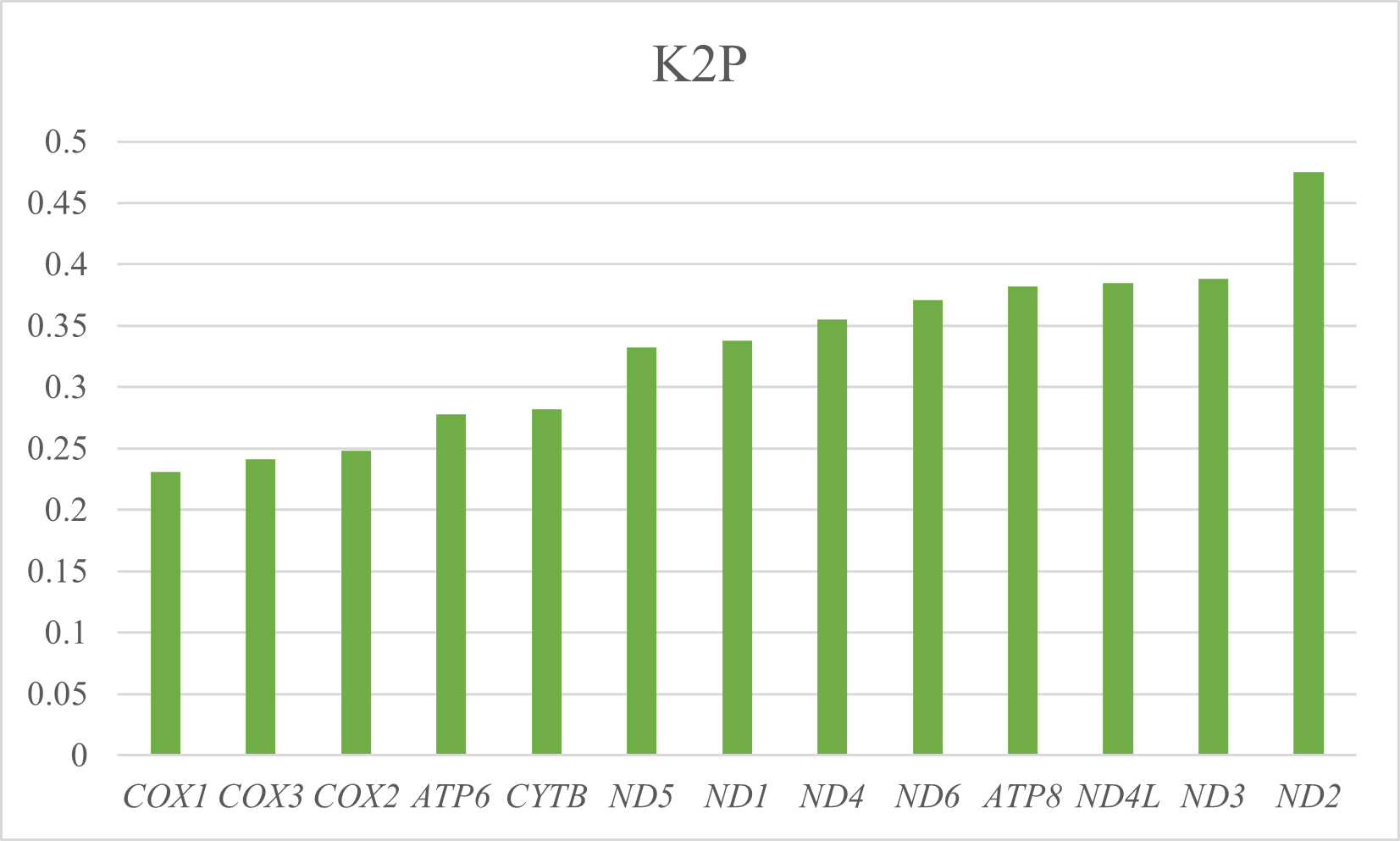

Supplement: Supplementary file 1 [file animals-15-01493-s001.zip › Figure S1.tif]

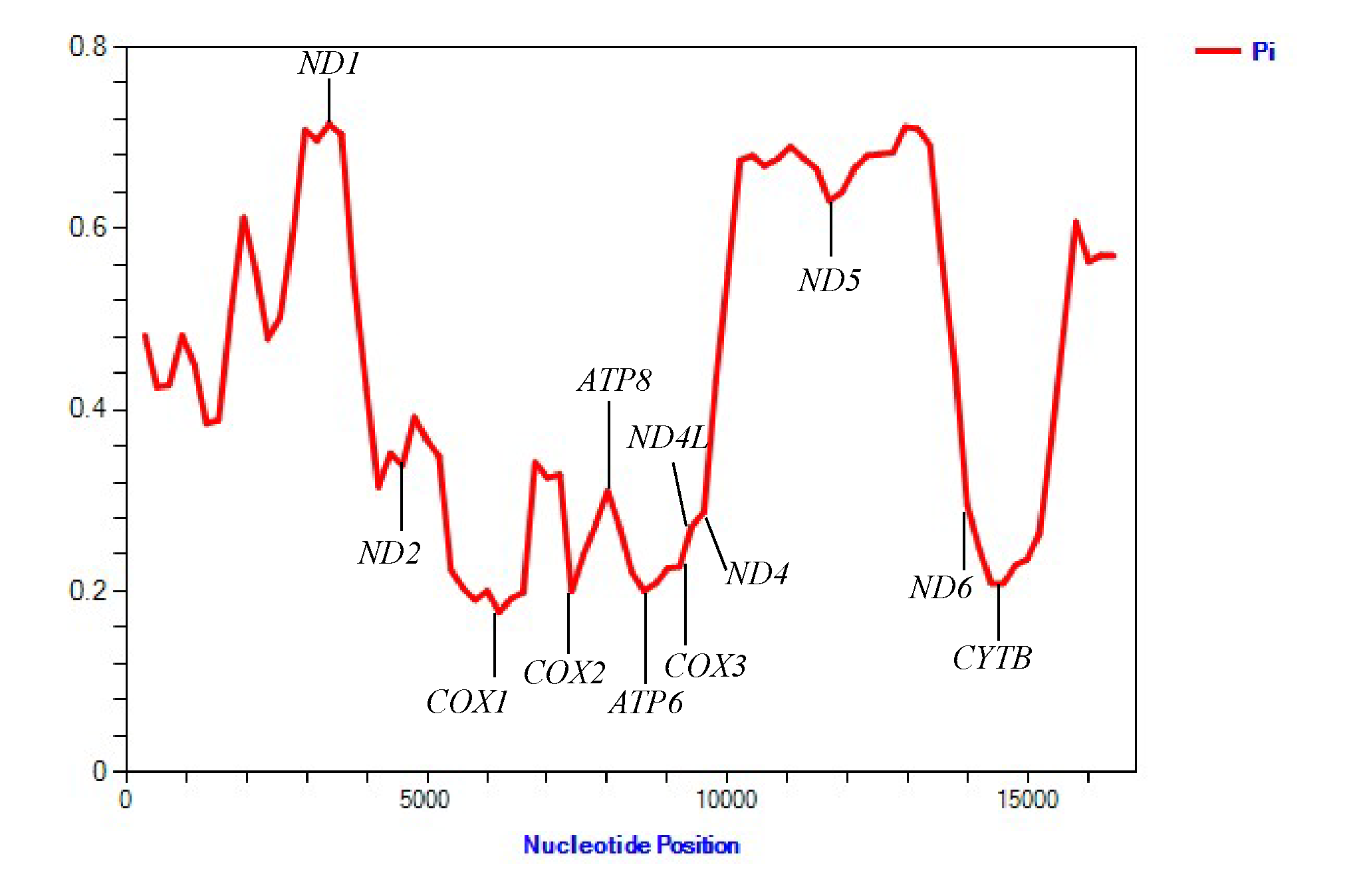

Supplement: Supplementary file 1 [file animals-15-01493-s001.zip › Figure S2.tif]

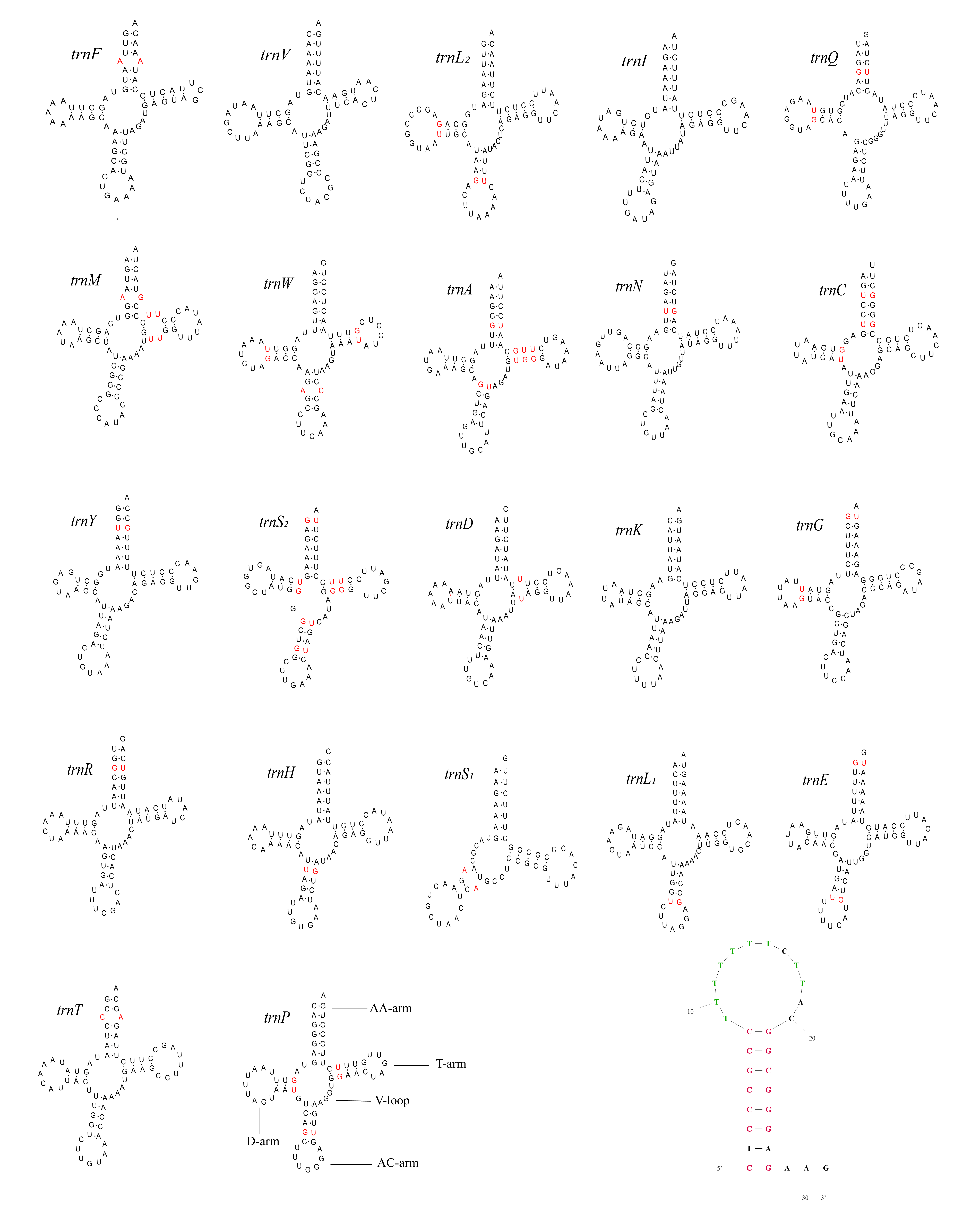

Supplement: Supplementary file 1 [file animals-15-01493-s001.zip › Figure S3.tif]

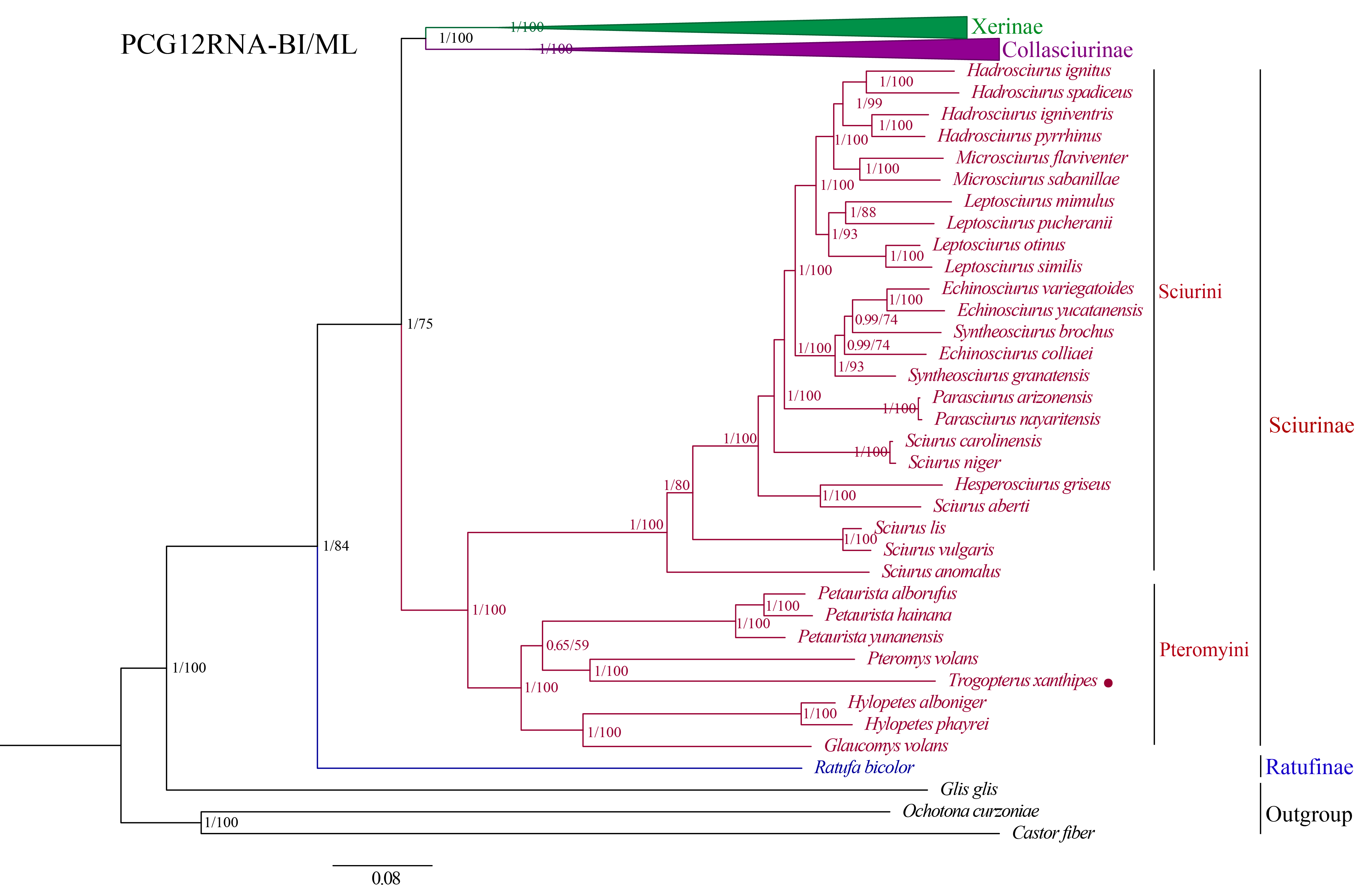

Supplement: Supplementary file 1 [file animals-15-01493-s001.zip › Figure S4.tif]

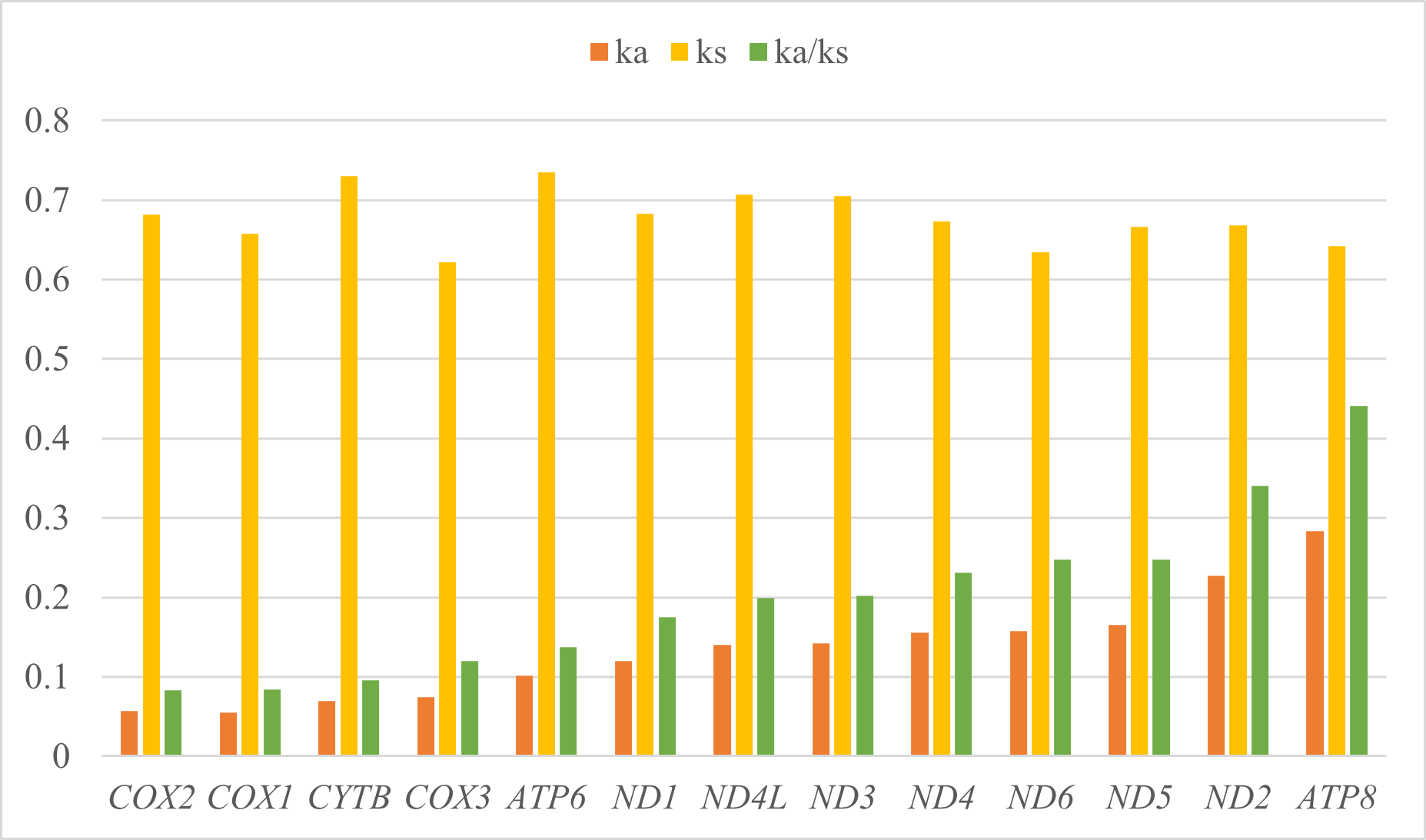

Supplement: Supplementary file 1 [file animals-15-01493-s001.zip › Figure S6.tif]
